# Underestimation of Stent Efficacy: Untreated Artery Events Distort Coronary Intervention Outcomes

**DOI:** 10.1002/clc.70229

**Published:** 2025-12-05

**Authors:** Mauricio Rigodanzo Mocha, Whady Hueb, Paulo Cury Rezende, Matheus de Oliveira Laterza Ribeiro, Thiago Luis Scudeler, Eduardo Gomes Lima, José Antonio Franchini Ramires, Roberto Kalil Filho

**Affiliations:** ^1^ Divisão Clinica, Instituto do Coracao, Hospital das Clinicas HCFMUSP, Faculdade de Medicina Universidade de São Paulo Sao Paulo BR

**Keywords:** angioplasty, cardiovascular events, percutaneous coronary intervention, stable coronary artery disease

## Abstract

**Background:**

Evaluation of percutaneous coronary intervention (PCI) effectiveness typically focuses on overall coronary events, often overlooking the specific location of these events within the coronary vasculature. This study aimed to evaluate the incidence of coronary events in patients treated with PCI and to investigate the correlation between these events, the location of the event within the treated site, and the type of stent used.

**Methods:**

This study determined the proportion of coronary events occurring after percutaneous coronary intervention (PCI) and investigated the association between these events and the location of the previous treatment site. A retrospective cohort analysis was conducted on patients with stable coronary artery disease (CAD) who underwent PCI using either conventional or drug‐eluting stents. Data was obtained from the MASS Clinical Research Unit database. The location of the coronary event was determined by angiography.

**Results:**

Five hundred and sixty two patients were included, with 232 (41.3%) experiencing coronary events. 55.8% of the events occurred at the treated site, while the remaining 44.2% were unrelated to the treated site. Of the events related to the treated site, 54.1% occurred in the conventional stent group and 61.1% in the drug‐eluting stent group. No statistically significant association was found between the event in the treated site and the type of stent used (*p* = 0.363).

**Conclusions:**

A significant proportion of coronary events occurred in untreated sites. Our results suggest that isolated evaluation of the treated site may provide a more accurate estimate of the effectiveness of stents.

## Abstract (highlights)

**What Is Already Known on This Topic:**

- PCI studies typically evaluate coronary events globally, without specifying the exact location in the treated vessel.

**What This Study Adds:**

- This study shows that many events occur outside the treated site and that stent type is not significantly related to events in the treated vessel.

**How This Study Might Affect Research, Practice, or Policy:**

- The results suggest that the effectiveness of stents should be reevaluated, also considering events outside the treated sites.

## Graphical abstract

This prospective study evaluated 562 patients with stable coronary disease undergoing PCI. It was observed that 55.8% of events occurred in the treated vessel and 44.2% in untreated vessels. There was no significant difference between bare‐metal and drug‐eluting stents. These findings reinforce the importance of analyzing events by treated vessel.

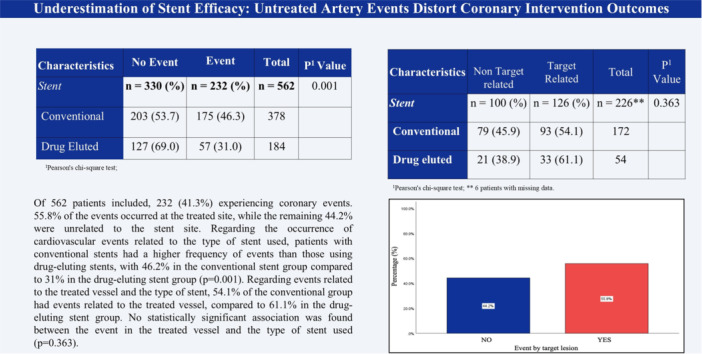

**Abbreviations**
ACC: American College of Cardiology
AHA: American Heart Association
AMI: acute myocardial infarction
BMI: body mass index
CABG: coronary artery bypass graft
CAD: coronary artery disease
CCS: Canadian Cardiovascular Society
EF: ejection fraction
FMUSP: Faculdade de Medicina da Universidade de São Paulo
HbA1c: glycated hemoglobin
HDL: high‐density lipoprotein
IVUS: intracoronary vascular ultrasound
LDL: Low‐density lipoprotein
MACE: major adverse cardiac events
MASS: Medicine, Angioplasty, or Surgery Study
MI: myocardial infarction
PCI: percutaneous coronary intervention
SCAI: Society for Cardiovascular Angiography and Interventions
SD: standard deviation
SS: syntax score
OCT: optical coherence tomography
UA: unstable angina

## 1 Introduction

Percutaneous coronary intervention (PCI) is a therapeutic modality widely used in the management of coronary artery disease (CAD), providing a less invasive alternative compared to coronary artery bypass graft surgery [1, 2]. Although several studies have investigated the effectiveness of PCI, much of this study uses a composite of adverse events known as MACE (Major Adverse Cardiac Events) to evaluate results [3, 4, 5, 6]. MACE encompasses events such as death, myocardial infarction, new interventions and stroke. However, the use of MACE as an indicator of PCI success may not reliably reflect the real benefits and possible limitations of percutaneous treatment. This occurs because the adverse events considered in MACE are not always directly related to the target site treated [5, 6]. Other studies attempting more precise evaluations often correlate these events with the treated vessel [7]. For example, death may result from causes not associated with percutaneous treatment; myocardial infarction can occur in an untreated coronary site; and new interventions may not be necessary due to factors related to the initial procedure. Furthermore, cerebrovascular accident (CVA) does not have a direct connection with coronary events, which questions its inclusion in the evaluation of percutaneous coronary treatment.

Given these limitations, this study proposes an analysis centered exclusively on the treated site, focusing on adverse events directly attributable to it. We believe this approach will provide a more accurate and detailed understanding of the results of percutaneous treatment, allowing for a more genuine assessment of its efficacy and safety. By excluding events that are not directly related to the treated site, we seek to provide more specific and relevant data that can guide clinical practices and future research more effectively.

## 2 Methods

This study comprises a retrospective cohort analysis of patients diagnosed with stable coronary artery disease (CAD) who underwent PCI using either conventional or drug‐eluting stents. Data from the MASS Clinical Research Unit database, known as the Medicine, Angioplasty, or Surgery Study were utilized, including outpatient individuals from the Heart Institute of the University of São Paulo, Brazil.

Inclusion criteria encompassed coronary artery disease, preserved left ventricular function, and a formal indication for PCI. All eligible patients provided informed consent as per the protocol established by the MASS Research Unit and were treated in compliance with the Helsinki Declaration.

Severe coronary obstruction was defined as visually estimated stenosis equal to or greater than 70% in epicardial vessels. Outcomes were assessed according to the location of the treated vessel (target site) and the type of stent used. Post‐event angiograms determined whether the event was related to the target site, considering occurrences within the stent or up to 5 mm proximal/distal to the edges. Lesions were classified according to the AHA/ACC criteria (extension, calcification, tortuosity, and location). Angiographic review was performed by two independent interventional cardiologists, blinded to the stent type and clinical outcome.

Exclusion criteria included patients with acute coronary syndrome, urgent myocardial revascularization indication, severe left ventricular dysfunction, contraindication to any components of clinical treatment, anatomically significant valvular disease, chronic kidney disease (creatinine ≥ 2.0 mg/dL), active rheumatologic disease, sepsis, pulmonary embolism, or deep vein thrombosis within the last 6 months, and malignancy under treatment.

To assess procedural outcomes, we conducted a targeted analysis based on the location of the treated vessel (target site) and the type of stent used. Post‐event angiograms were utilized to determine if the event was related to the target site. Events were considered related to the target site if they occurred within the stented segment and extended up to 5 mm proximal and distal to the stent edges [8]. Additionally, adjusted analysis calculations were performed to eliminate potential confounding factors such as age, gender, body mass index (BMI), hypertension, smoking, glomerular filtration rate, LDL, HDL, triglycerides, fasting glucose, glycosylated hemoglobin, left ventricular function (EF), and limiting angina. Furthermore, the SYNTAX Score (SS) I and II calculation was employed for a more precise evaluation of atherosclerotic burden and clinical variables [9, 10]. The residual SYNTAX score was also calculated to assess whether the atherosclerotic burden after the initial procedure could be a confounding factor in the analysis of the results.

Each patient was evaluated by the medical team comprising clinical cardiologists from the MASS Clinical Research Unit outpatient clinic. All patients received institution‐prescribed medications in accordance with current guidelines, and the same criteria were applied for associated morbidities. The choice of stent depended on the research protocol and, in the absence of prior specification, on the choice of the interventional cardiologist and the availability in the service. Following the procedure, all patients underwent routine follow‐up with periodic outpatient consultations and procedures as per current guidelines. Patients were categorized based on whether they experienced any of the following events: fatal or nonfatal myocardial infarction (MI), unstable angina (UA), repeat revascularization (PCI or CABG), or angiographic restenosis (obstructions exceeding 70%) [11]. Restenosis was included even if it did not require further intervention. These outcomes were then analyzed according to the treated artery site (target site) and the type of stent used.

### 2.1 Statistical Analysis

For statistical analysis, quantitative variables were presented as means, medians, standard deviations, and the 25th and 75th percentiles. Absolute and relative frequencies were calculated for qualitative variables. The association between qualitative variables was assessed using Pearson's chi‐square test or Fisher's exact test when appropriate. Normality of quantitative data was tested using the Kolmogorov–Smirnov test. The non‐parametric Mann–Whitney test was used to compare a quantitative variable between two groups. A significance level of 5% was adopted. The analyses were conducted using the statistical software SPSS for Windows v.25.

## 3 Results

**Figure 1:**
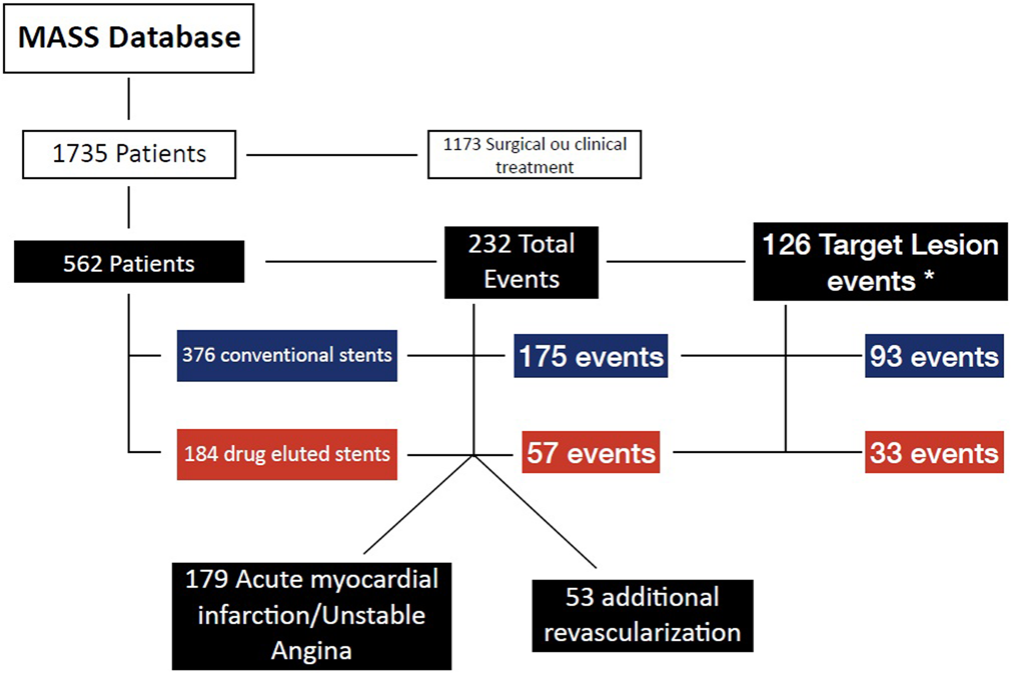
Flow Diagram from patients' data.

Between 1997 and 2018, all patients diagnosed with stable coronary artery disease and referred to the MASS unit at the Heart Institute of the Hospital das Clínicas, FMUSP, who were included in the research protocols and underwent angioplasty, were considered for this study. Of the 1735 patients analyzed, 562 met the inclusion criteria. Of these, 232 (41.3%) experienced at least one of the pre‐defined adverse events. (Figure 1).

The baseline characteristics of the population are outlined in Table 1. The mean (SD) age of the population was 59.8 years, with a majority being male (66%). The mean body mass index (BMI) was 27.8, indicating overweight status. Among the included patients, 43.9% had a history of previous myocardial infarction (MI) at the beginning of follow‐up, 83.8% were hypertensive, 52.1% were diabetic, and 49.8% had never smoked.

**Table 1:** Baseline characteristics of the study population.

Characteristics	Event		Total	*p* value
	No	Yes		
	*n* = 330	*n* = 232	*n* = 562	
Demographic Profile				
Male (%)	216 (65.4)	155 (66.8)	371 (66)	0.738^a^
Age Mean (SD) years	61.2 (8.2)	57.9 (9.2)	59.8 (8.8)	< 0.001^b^
BMI (kg/m^2^) (SD)	27.83 (4.3)	27.83 (4.4)	27.83 (4.4)	0.885^b^
Medical history				
Previous myocardial infarction (%)	131 (39.6)	116 (50.0)	247 (43.9)	0.015^a^
Hypertension (%)	272 (82.4)	199 (85.7)	471 (83.8)	0.288^a^
Type 2 diabetes (%)	170 (51.5)	123 (53.0)	293 (52.1)	0.726^a^
Never dmoke	171(51.8)	109 (46.9)	280 (49.8)	0.428^a^
Angina				
CCS I (%)	51 (15.4)	16 (6.8)	67 (11.9)	
CCS II (%)	146 (44.2)	101 (43.5)	247 (43.9)	
CCS III (%)	90 (27.2)	85 (36.6)	175 (31.1)	
CCS IV (%)	43 (13.0)	30 (12.9)	73 (12.9)	
Ischemia^c^ (%)	122 (43.8)	70 (25.1)	192 (69.0)	0.298^a^
Ejection fraction mean %, (SD)	62.7 (8.8)	60.2 (9.9)	61.6 (9.3)	0.005^b^
Laboratorial findings				
LDL, mean (SD), mg/dL	120.1 (42.3)	126.9 (46.2)	122.9 (44.0)	0.14^b^
HbA1c (SD)	6.8 (1.7)	7.0 (1.9)	6.9 (1.8)	0.276^b^
Creatinina mg/dL (SD)	1.1 (0.3)	1.0 (0.3)	1.0 (0.3)	0.294^b^
Angiographic findings				
Uni‐vessel disease, No.(%)	2 (0.6)	2 (0.8)	4 (0.7)	
Double‐vessel disease, No. (%)	144 (43.6)	93 (40.0)	237 (42.1)	
Triple‐vessel disease, No. (%)	184 (55.7)	137 (59.0)	321 (57.1)	
Syntax I (SD)	16.84 (7.19)	16.77 (6.51)	16.81 (6.9)	0.971^b^
Syntax II (SD)	28.64 (7.9)	27.47 (8.5)	28.18 (8.2)	0.1^b^
Syntax residual (SD)	7.50 (6.5)	7.67 (6.4)	7.57 (6.5)	0.745^b^
Stent number (SD)	2.1 (1.1)	2.0 (1.1)	2.1 (1.1)	0.238^b^
Drug eluted stents (%)	127 (38.4)	57 (24.5)	184 (32.7)	
Conventional stents (%)	203 (61.5)	175 (75.4)	378 (67.2)	

*Note:* Baseline Characteristics of the Study Population, by Event Occurrence. This table presents the demographic, medical history, angina, ischemia, ejection fraction, laboratory findings, and angiographic findings of the study population, categorized by the occurrence of events.

^a^ Pearson's chi‐square test.

^b^ Mann–Whitney test; SD: standard deviation.

^c^ Information for 278 patient.

Table 1 also presents information about ischemia and coronary anatomy, which are crucial for understanding the context of this study. Most patients had multi‐vessel disease (42.1% with two‐vessel disease and 57.1% with three‐vessel disease). Notably, 92% of the patients were involved in the left anterior descending artery. Noninvasive ischemia testing was available for 278 out of 562 patients, with 69% showing evidence of ischemia. All patients presented with some degree of angina according to the Canadian Cardiovascular Society (CCS) classification, with 75.0% experiencing CCS II or III angina. Table 1 also includes laboratory data and left ventricular ejection fraction.

Due to the anatomical complexity of coronary artery disease and considerable interindividual variation, which may lead to difficulties in performing angioplasty and introduce confounding factors and biases in event analysis, we chose to calculate the Syntax score (SS) for these patients, which is also present in Table 1. No statistical difference was observed between the distributions of different Syntax scores (Syntax, Syntax 2, Syntax R) and the occurrence of events.

**Figure 2:**
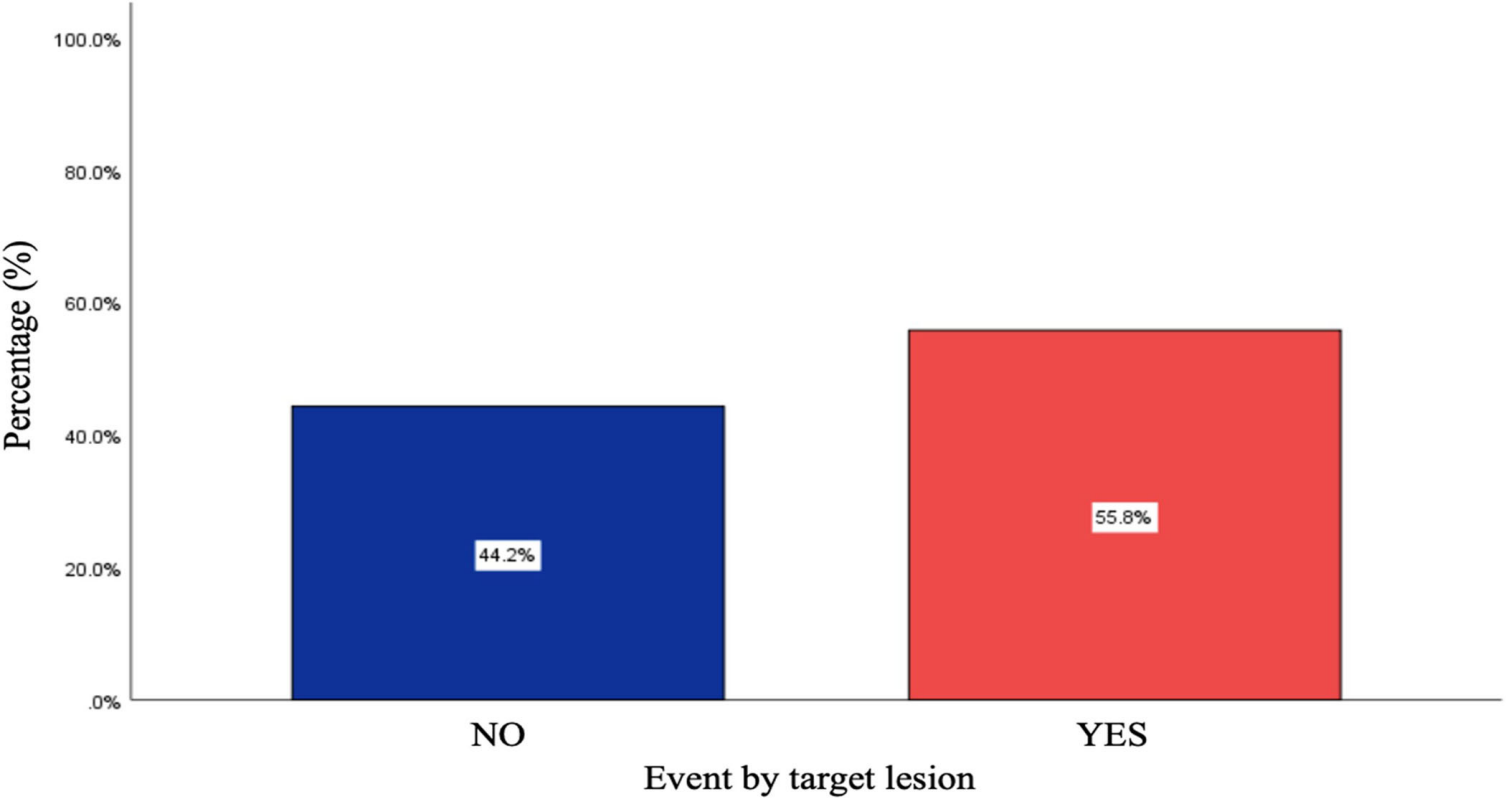
Event by target lesion—The column chart shows that 55.8% of the events occurred at the target lesion.

Of the 232 patients who experienced an event, 55.8% were related to the previously treated site (Figure 2). The most common events were acute myocardial infarction (AMI) and unstable angina (UA), accounting for 77.2% of all events, while repeat revascularization was necessary in 22.8% of cases (Table 2). Six patients died due to AMI, but it was not possible to determine from the available data whether these events were related to the previously treated site.

**Table 2:** Events related to the target vessel and type of all events.

Characteristics		Total
Total of events		***n*** = **232 (%)**
Target lesion related		126 (55.8)^a^
Type of event	AMI/UA	179 (77.2)
	Additional revascularization ATC/CABG	53 (22.8)

*Note:* Coronary Events by Category. This table shows the distribution of different types of coronary events (acute myocardial infarction, unstable angina, revascularization) experienced by the study population.

^a^ Six patients with missing data.

Regarding the occurrence of cardiovascular events related to the type of stent used, patients with conventional stents had a higher frequency of events than those using drug‐eluting stents, with 46.2% in the conventional stent group compared to 31% in the drug‐eluting stent group (*p* = 0.001), as shown in Table 3.

**Table 3:** Type of stent used related to event.

	Event			
	**NO**	**YES**	Total	
Characteristics	***n*** = **330 (%)**	***n*** = **232 (%)**	***n*** = **562**	*p* a value
*Stent*				0.001
Conventional	203 (53.7)	175 (46.3)	378	
Drug Eluted	127 (69.0)	57 (31.0)	184	

*Note:* Coronary Events by Stent Type. This table presents the frequency of coronary events in patients who received conventional stents versus drug‐eluting stents.

^a^ Pearson's chi‐square test.

Regarding events related to the treated site and the type of stent, 54.1% of the conventional group had events related to the treated site, while patients with drug‐eluting stents who had events related to the treated site were 61.1%. However, no statistically significant association was found between the event related to the treated site and the type of stent used (*p* = 0.363), as shown in Table 4.

**Table 4:** Event related to the target vessel by type of stent.

	Target lesion related			
	No	Yes	Total	
Characteristics	*n* = 100 (%)	*n* = 126 (%)	*n* = 226^b^	*p* a value
*Stent*				0.363
Conventional	79 (45.9)	93 (54.1)	172	
Drug eluted	21 (38.9)	33 (61.1)	54	

*Note:* Association Between Event Location and Stent Type. This table examines the relationship between the location of the coronary event (treated vessel vs. untreated vessel).

^a^ Pearson's chi‐square test.

^b^ Six patients with missing data.

The sample allows us to verify that there are no statistically significant differences between event‐free survival curves according to the stent used and according to the related event.

**Figure 3:**
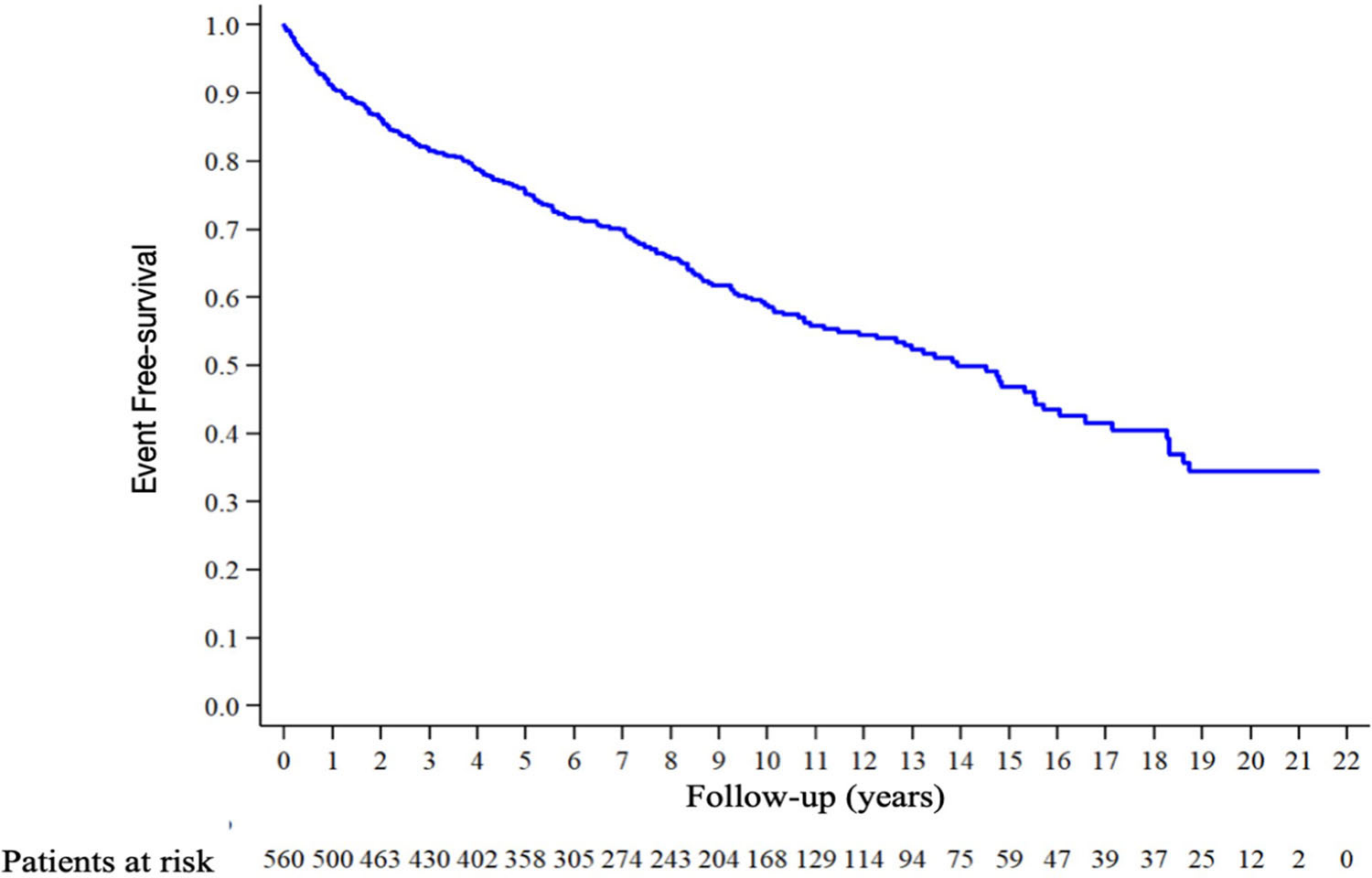
Event‐free survival—Kaplan–Meier curve that illustrates the probability of remaining free from events over a 22‐year period.

For event‐free survival, it was observed that 58.7% of patients are free from events at 10 years. The event‐free and overall survival curves are presented in Table 5 and Figure 3.

**Table 5:** Event‐free survival.

	Event	Mean (SD)	Median	Probability				*p* a value
		years	years	1‐ano	2‐anos	5‐anos	10‐anos	
Event‐free survival								
General (%)	232 (41.3)	12.7 (0.4)	(13.9)	(90.5)	(85.9)	(75.3)	(58.7)	
Stent								0.172
Conventional stents (%)	175 (46.3)	12.4 (0.5)	12.8	(90.2)	(85.4)	(74.7)	(55.6)	
Drug eluted stents	57 (31.0)	11.2 (0.5)	15.3	(91.1)	(87.0)	(76.7)	(65.5)	

*Note:* Event‐Free and Overall Survival. This table provides data on event‐free survival and overall survival rates at different time points, allowing for an assessment of long‐term outcomes.

^a^ Log‐rank test.

In the analysis of overall survival, it is concluded that, generally, patients have a 10‐year survival rate of 84.7%. Numbers that are expected for patients with initially stable coronary artery disease with preserved ejection fraction.

In the multivariate analysis, events were categorized as either treated vessel‐related or nontreated vessel‐related. The following variables were used as predictors: baseline and residual SYNTAX scores, glycated hemoglobin levels, LDL cholesterol levels, stent type, and the occurrence of previous myocardial infarction.

**Figure 4:**
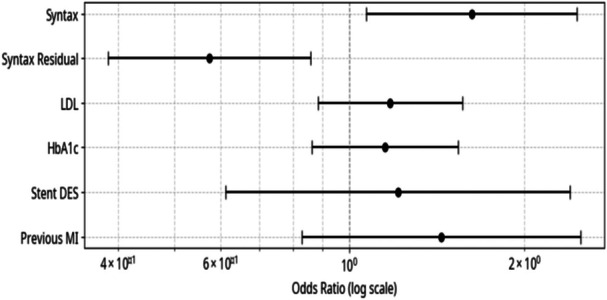
Vessel Site Related X No Vessel Site Related Events—Forest Plot.

We observed that the SYNTAX score had a statistically significant influence. Higher SYNTAX scores correlated with more complex angioplasty procedures and the use of a greater number of stents. Similarly, an elevated residual SYNTAX score, indicating more unaddressed lesions, increased the risk of an event being correlated with a site not previously treated. The remaining predictors did not demonstrate statistical significance. Forest plot illustrating the adjusted Odds Ratios and 95% confidence intervals for predictors of treated vessel‐related events in the multivariate analysis. HbA1c: Glycated Hemoglobin; LDL: Low Density Lipoprotein; MI: Myocardial Infarction; Stent DES: Drug‐Eluting Stent; Syntax: SYNTAX Score. SYNTAX and Residual SYNTAX scores are statistically significant predictors of events. A high SYNTAX score increases the risk of events at the treated site, while a high Residual SYNTAX score shits that risk toward the nontreated vessel sites, as illustrated in Figure 4.

## 4 Discussion

Coronary artery disease (CAD), characterized by its multifactorial course and anatomical heterogeneity, remains a challenge in defining effective therapeutic strategies, especially in patients with stable disease. Recent studies, such as the ISCHEMIA Trial [12], have revealed.

That early coronary intervention does not significantly reduce mortality or the occurrence of major cardiovascular events when compared to the optimized clinical strategy. However, it is important to emphasize that such studies use broad endpoints, often based on events occurring in any coronary territory, regardless of the vessel treated. Furthermore, they consider death from any cause, unplanned coronary intervention, or stroke. This broad approach can limit the accurate assessment of the specific efficacy of percutaneous intervention.

Our study proposes a different and more specific approach: evaluating coronary events directly related to the treated vessel site. We found that 44.2% of events occurred in untreated sites, suggesting that a substantial portion of post‐PCI coronary outcomes cannot be attributed to the initial procedure. This distinction is crucial, as it allows us to better understand the direct impact of angioplasty on the target territory.

On the other hand, among the 55.8% of events related to the treated vessel, it is important to consider that not all reflect procedural failure. This is due to the possibility of coronary disease progression even in these territories, especially when evaluating long‐term outcomes, despite the local changes generated by the presence of the stent, including chronic inflammatory mechanisms [13, 14, 15]. The occurrence of in‐stent restenosis, disease progression in adjacent segments, or neo atherosclerosis may explain these events, even in the case of a technically successful procedure. Unfortunately, the lack of intravascular imaging modalities (intravascular ultrasound or optical coherence tomography) in our sample limited the ability to accurately distinguish these causes. We acknowledge this limitation as relevant and emphasize that the use of intracoronary imaging could have improved the interpretation of events in the treated area.

Stent type also deserves consideration. Although the group with drug‐eluting stents showed a trend toward a lower incidence of events related to the treated vessel compared to the bare‐metal stent group, this difference was not statistically significant. This indicates that, from a target lesion perspective, the superiority of drug‐eluting stents may be less pronounced than aggregate data generally suggest. This finding is in line with the results of the NORSTENT study [16], which also found no significant difference in mortality or infarction between the stent types, despite the lower revascularization rate with drug‐eluting stents.

The main contribution of this study is to propose a methodological refinement in the evaluation of PCI: by distinguishing between events related and unrelated to the treated vessel, we bring the analysis closer to the true impact of the procedure on the target lesion. This strategy can reduce common biases in studies using global outcomes, avoiding both overestimation of the event rate and underestimation of the benefit of PCI.

However, it is important to note that our study included drug‐eluting stents of different generations, which may influence the results. Newer‐generation stents tend to have better clinical performance, and this heterogeneity constitutes a limitation that prevents definitive conclusions regarding the specific efficacy of each device type. Therefore, we believe that analysis focused on the treated vessel represents a more accurate approach to measuring the true effectiveness of PCI. We recommend that subsequent studies, especially those with a prospective design, adopt this type of stratification, which may improve the assessment of the benefits of percutaneous intervention and better guide therapeutic decision‐making. The most relevant aspect of our analysis is that much of the current literature continues to evaluate stent efficacy by considering global events—such as all‐cause mortality or infarction in any coronary artery—which can lead to an overestimation of the event rate and, consequently, an underestimation of the true benefit of the intervention. By including events unrelated to the treated artery, the ability to isolate the effect of treatment on the target lesion is lost. Our proposal, by differentiating between events related and unrelated to the treated vessel, contributes to a more reliable assessment of stent efficacy and may help refine outcome criteria in future clinical trials.

### 4.1 Study Limitations

This study has limitations that should be considered. First, the lack of intravascular imaging (e.g., IVUS or OCT) limited a more precise understanding of the mechanisms underlying events related to the treated vessel, such as in‐stent restenosis, disease progression, or neoatherosclerosis. These tools would have improved the assessment of the efficacy of local intervention. Furthermore, although we observed a trend toward fewer events with drug‐eluting stents (DES) compared to bare‐metal stents (BMS), this analysis was limited by the heterogeneity of the DES generations included, which differ in drug type, platform, and biocompatibility. Because newer DES generations have demonstrated improved safety and efficacy, their combined analysis with older devices introduces bias and prevents definitive conclusions about individual stent performance. The retrospective, observational study design, despite its novel focus on treated versus untreated vessels, is subject to selection bias and residual confounding factors. Furthermore, the lack of standardized clinical follow‐up may have affected event detection. Finally, the single‐center design, while limiting generalizability, ensures uniform diagnostic and therapeutic protocols, reducing variability and enhancing internal validity through consistent data collection and team expertise. These limitations reinforce the need for prospective studies using uniform stents and intracoronary imaging to confirm and expand upon our findings.

## 5 Conclusions

This study's findings suggest a need to re‐evaluate the criteria used to measure stent effectiveness. A more precise approach should clearly distinguish between events related and unrelated to the treated lesion.

Our cohort analysis demonstrated that when events were evaluated without considering their location, patients initially treated with conventional stents had higher event rates. However, when analyzed in relation to the treated site, no statistically significant difference was observed between stent types.

These findings have important implications for clinical practice and research. Accurately assessing stent efficacy is crucial for making informed treatment decisions and optimizing patient care. Future studies should adopt a more targeted approach to event analysis, focusing on events directly attributable to the treated site to provide a clearer understanding of the true benefits of different stent technologies and guide the development of more effective interventions for coronary artery disease.

## Conflicts of Interest

The authors declare no conflicts of interest.

## Data Availability

All data generated or analyzed during this study are included in this published article and its supporting information files and are freely available on the database.
